# Processes and Outcomes of the Veterans Health Administration Safe Patient Handling Program: Study Protocol

**DOI:** 10.2196/resprot.2905

**Published:** 2013-11-18

**Authors:** Deborah Rugs, Peter Toyinbo, Nitin Patel, Gail Powell-Cope, Bridget Hahm, Christine Elnitsky, Karen Besterman-Dahan, Robert Campbell, Bryce Sutton

**Affiliations:** ^1^United States Veterans Health AdministrationCenter of Innovation on Disability & Rehabilitation ResearchJames A Haley Veterans HospitalTampa, FLUnited States; ^2^School of NursingCollege of Health and Human ServicesUniversity of North Carolina at CharlotteCharlotte, NCUnited States

**Keywords:** back injuries, occupational injuries, moving and lifting patients, methods, program evaluation

## Abstract

**Background:**

Health care workers, such as nurses, nursing aides, orderlies, and attendants, who manually move patients, are consistently listed in the top professions for musculoskeletal injuries (MSIs) by the Bureau of Labor Statistics. These MSIs are typically caused by high-risk patient caregiving activities. In 2008, a safe patient handling (SPH) program was implemented in all 153 Veterans Administration Medical Centers (VAMCs) throughout the United States to reduce patient handling injuries.

**Objective:**

The goal of the present study is to evaluate the effects associated with the national implementation of a comprehensive SPH program. The primary objectives of the research were to determine the effectiveness of the SPH program in improving direct care nursing outcomes and to provide a context for understanding variations in program results across sites over time. Secondary objectives of the present research were to evaluate the effectiveness of the program in reducing direct and indirect costs associated with patient handling, to explore the potential mediating and moderating mechanisms, and to identify unintended consequences of implementing the program.

**Methods:**

This 3-year longitudinal study used mixed methods of data collection at 6- to 9-month intervals. The analyses will include data from surveys, administrative databases, individual and focus group interviews, and nonparticipant observations. For this study, a 3-tiered measurement plan was used. For Tier 1, the unit of analysis was the facility, the data source was the facility coordinator or administrative data, and all 153 VAMCs participated. For Tier 2, frontline caregivers and program peer leaders at 17 facilities each completed different surveys. For Tier 3, six facilities completed qualitative site visits, which included individual interviews, focus groups, and nonparticipant observations. Multiple regression models were proposed to test the effects of SPH components on nursing outcomes related to patient handling. Content analysis and constant comparative analysis were proposed for qualitative data analysis to understand the context of implementation and to triangulate quantitative data.

**Results:**

All three tiers of data for this study have been collected. We are now in the analyses and writing phase of the project, with the possibility for extraction of additional administrative data. The focus of this paper is to describe the SPH program, its evaluation study design, and its data collection procedures. This study evaluates the effects associated with the national implementation of a comprehensive SPH program that was implemented in all 153 VAMCs throughout the United States to reduce patient handling injuries.

**Conclusions:**

To our knowledge, this is the largest evaluation of an SPH program in the United States. A major strength of this observational study design is that all VAMCs implemented the program and were included in Tier 1 of the study; therefore, population sampling bias is not a concern. Although the design lacks a comparison group for testing program effects, this longitudinal field study design allows for capturing program dose-response effects within a naturalistic context. Implementation of the VA-wide SPH program afforded the opportunity for rigorous evaluation in a naturalistic context. Findings will guide VA operations for policy and decision making about resources, and will be useful for health care, in general, outside of the VA, in implementation and impact of an SPH program.

## Introduction

### Background

Health care workers, such as nurses, nursing aides, orderlies, and attendants, who manually move patients, are listed in the top professions for musculoskeletal injuries (MSIs) by the Bureau of Labor Statistics [1]. MSIs are typically caused by high-risk patient caregiving activities that include turning and repositioning, lateral transfers, assisting to standing position, exiting the bed, and transferring from one surface to another, such as from a bed to chair. Manual patient handling has a deleterious effect on staff, patient safety, and organizational factors such as nursing staff turnover, job satisfaction, and cost due to workers compensation and lost work time. Indirect costs associated with MSIs include replacing employees (workforce attrition), injury investigation time, supervision time, training, staff morale, disruptions in teamwork and workflow, administrative time, and paid overtime [2,3].

Safe patient handling (SPH) programs consist of the following components: equipment, ergonomic assessment protocols, no-lift policies, and staff training. Recent studies have shown that SPH programs have a positive effect on MSIs [4-11]. In one study, it was observed that injury rates dropped as much as 73% after implementing a SPH program [10].

Given that the Veterans Health Administration (VHA) employs approximately 77,000 nurses, nursing assistants, health aids and technicians, and trainees [12], the effect of patient handling injuries on individuals and on the system is substantial. In 2008, the VA implemented a SPH program in all 153 Veterans Administration Medical Centers (VAMCs) throughout the United States. The implementation of the SPH program was financially supported by an investment of US $205 million. The National VA SPH program funded local equipment purchases, facility coordinators, and technical guidance from a VA expert in SPH and ergonomics. This paper reports on the study protocol for the mixed methods evaluation of the national implementation of the VHA SPH program. Many of the measures and evaluation procedures reported here were used in prior local and regional VHA studies [13,14].

### Objectives

The goal of the research was to assess the processes and outcomes associated with the implementation of a comprehensive SPH program across all 153 VAMCs. The primary objectives of the research were to: (1) determine the effectiveness of the SPH program in improving direct care nursing outcomes (eg, incidence and severity of injuries and job satisfaction) and patient outcomes (injuries associated with patient handling), and (2) provide a context for understanding variations in program results across multiple facilities over time including barriers and facilitators to implementation, local customization of the program, and organizational and individual factors that influenced implementation and program effects. Secondary objectives of the research were to: (1) evaluate the effectiveness of the program in reducing direct and indirect costs associated with patient handling, (2) explore the potential mediating and moderating mechanisms (eg, strength of program implementation and program uptake) by which SPH program exerts its effects on outcomes, and (3) identify unintended consequences of implementing the program. All data for this study have been collected. We are now in the analyses and writing phase of the project, with the possibility for extraction of additional administrative data. The intention of this paper is to describe the SPH program, its evaluation study design, and its data collection procedures.

### Theoretical Framing

Implementation of evidence-based practices is a function of multiple factors including the nature of the evidence, the transmission of knowledge to users, and context of implementation including the health system and organizational factors [15]. Theoretical and conceptual models commonly used to either guide implementation or account for these factors include Diffusion of Innovations [16], Stetler Model [17], Translating Research into Practice [18], and Promoting Action on Research Implementation in Health Services [19].

The SPH program was primarily conceptualized as an evidence-based health care safety program based on ergonomic principles and designed to improve both caregiver and patient outcomes. The key elements of SPH program include: (1) ergonomic risk assessment of each unit or area to evaluate and address conditions that may present injury risks related to patient handling, (2) selection and purchase of safe handling equipment, (3) training and continuing staff SPH competency evaluations, (4) ongoing evaluation and reporting of program outcomes, (5) peer leadership to implement at the unit level, (6) a multidisciplinary facility-level Safe Patient Handling Committee, representing all stakeholders of the program, (7) local policy mandating minimal patient lifting and moving, and (8) a part-time facility coordinator to assume leadership of the program implementation and serve as a bridge among administrators, managers, and caregivers.

**Figure 1:**
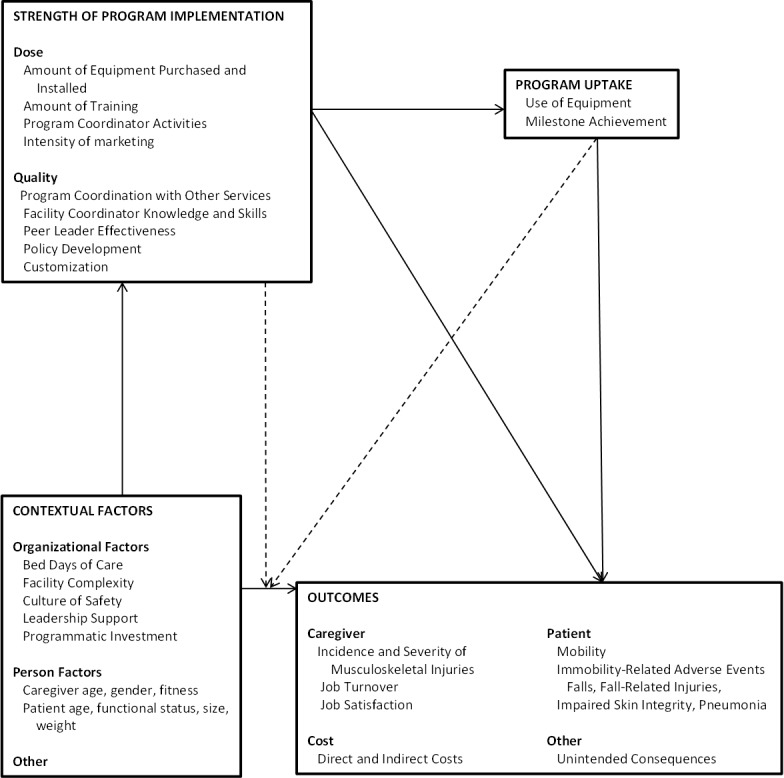
Conceptual framework for the implementation and evaluation of the VA Safe Patient Handling Program.

The selection of indicators for measuring context, knowledge transmission, program, and outcomes was guided by theoretical underpinnings of implementation, and all of these variables were grouped into relational categories in a quantitative conceptual model (see Figure 1). However, not all expected variables were measured due to limitations of the administrative data and availability of administrative data sets at the facility and patient levels. To set the framework for more specific hypotheses in subsequent sections, we specified four global hypotheses in general terms within the context of our quantitative conceptual model of patient-handling-related outcomes (eg, MSI incidence rates for nursing professions, job satisfaction, patient injury rates, and costs). The hypotheses are as follows: (H1) contextual factors will be used as significant risk factors (predictors) for the outcomes; (2) after accounting for contextual factors, higher strength levels of SPH program implementation and uptake will be significantly associated with the favorable outcomes; (3) the risk association between contextual factors and the outcomes will be moderated by the strength of SPH program implementation and uptake; and (4) higher strength of program implementation will increase program uptake that in turn will lead to favorable outcomes. More specific versions of each hypothesis are described later under appropriate sections.

## Methods

This 3-year longitudinal study used mixed methods of data collection at 6- to 9-month intervals: analysis of data from surveys, administrative databases, individual and focus group interviews, and nonparticipant observations. All measures of indicators and data sources are given in Table 1.

**Table 1:** The measurement plan.

Indicator		Data source
**Strength of program implementation**		
	Number of equipment purchased and installed	Facility coordinator questionnaires
	Amount of training	
	Program coordinator activities	
	Intensity of marketing	
	Program coordination with other services	
	Peer leader effectiveness	
	Policy development	
	Customization	
	Management skills of facility coordinators	Observation and interviews during the site visits
**Program uptake**		
	Use of equipment	Facility coordinator questionnaires Interviews during site visits
	Milestone achievement	Facility coordinator questionnaire
**Context of implementation: organizational factors**		
	Bed days of care	VA administrative data (Bed Cube, VHA Support Service Center)
	Facility complexity	
	Culture of safety	Observation and interviews during site visits
	Leadership and stakeholder support	Facility coordinator and peer leader questionnaires Observation and interviews during site visits
	Programmatic investment	Facility coordinator questionnaire
**Nursing outcomes**		
	Incidence of injuries related to patient handling	VA Administrative Data (Veterans Affairs Nursing Outcomes Database/Automated Safety Incident Surveillance and Tracking System)
	Job turnover	VA Administrative Data (VHA Support Service Center)
	Job satisfaction	Staff survey
	Patient immobility adverse events	Interviews during site visits
	Unintended consequences	Interviews during site visits

### Sample and Sampling

For the present study, a 3-tiered evaluation plan was used. For Tier 1, the data sources were facility coordinators and administrative data, and all 153 VAMCs participated. The coordinators at 12 facilities were responsible for 2 VAMCs. Therefore, the maximum number of surveys that could be collected was 141 (92.1%). Table 2 presents the sample size and data collection timeline. Data sources for Tiers 2 and 3 were VHA staff who implemented the SPH program and frontline caregivers, and included a sample of 17 and 6, respectively, of the total 153 VAMCs. For Tier 2, we originally planned to randomly select 1 VAMC from each of the 21 regional Veterans-Integrated Services Networks, but 11 Veterans-Integrated Services Networks or regions did not volunteer to participate. For all most non-participating regional Veterans-Integrated Services Networks, the reason was that no individual volunteered to serve as the local investigator. For the 11 nonparticipating regions, we were able to replace 7 regions with volunteer facilities. Of all the participating facilities, 10/17 (59%) were the original randomly selected facilities and 7/17 (41%) were replacement facilities. Table 3 presents sample sizes and data collection timeline for Tier 2.

Ethnography was used as the qualitative methodology of Tier 3. Ethnography is a qualitative research method that is used to engage with others and their practices to better understand their culture [20]. It does not test a formative theory to establish whether it is right or wrong but rather may expand a model, discover associations among domains or variables, or match expected results from the formative theory with those obtained during the data collection process [21]. Purposive sampling was used to identify Veterans Administration Medical Centers for Tier 3. We used data from the first wave of data collection to determine the degree of deployment of SPH program elements. One of these facilities withdrew and was replaced with another facility from the same region. All six participating facilities, also participated in tier 2 data collection. The summary of data collected in Tier 3 is presented in Table 4. Within each site visit, the goal was to capture a well-rounded view of the SPH program at the facility. The individual interviews and focus groups included participation from direct caregivers, SPH program peer leaders, nurse managers, the SPH committee, facility coordinator, and hospital administration. For the unit observations, facilities were asked to identify one stellar unit and one struggling unit. Photographs were taken of equipment and staff during the unit observations.

**Table 2:** Administration times and sample size of facility coordinator surveys for Tier 1.

	December 2008	June 2009	December 2009	June 2010	December 2010	June 2011
Milestone Questionnaire	141	141	141	139	135	128
Program Dose Survey	141	141	140	137	135	127
Program Status Report	141	141	140	137	135	127

**Table 3:** Tier 2 peer and staff surveys administration times and sample size.

	February 2010		July 2010		February 2011		July 2011	
	Number of sites	Number of surveys	Number of sites	Number of surveys	Number of sites	Number of surveys	Number of sites	Number of surveys
Staff Survey	10	988	14	1382	17	1643	14	1294
Peer Leader Survey	10	133	14	173	17	242	15	272

**Table 4:** Sample size for Tier 3 site visits.

	2009	2010	2011	Total^a^
Number of sites	3	6	6	6
Number of site visits	4	7	4	15
Number of participants	71	124	91	286
Number of individual interviews	18	31	15	64
Number of focus groups	12	18	18	48
Number of photographs	86	132	85	303

^a^Several sites were visited more than once per year.

### Measures

#### Tier 1

The Milestone Questionnaire was developed to track each facility’s progress in meeting predetermined quarterly activities of the program. The milestones were developed by the VHA Occupational Health Program Office and covered patient care ergonomics, patient handling equipment, SPH policy, patient assessment forms, algorithms, and peer leader development. For each of 36 milestone items, the respondent could answer any of the following: did not start this task, started this task but did not complete it, or have completed this task. Furthermore, facility coordinators were asked if they had updated a list of 10 key stakeholders groups. They were also asked if they had made progress in developing their facility’s SPH policy which should have included seven core program components.

The Program Dose Survey measured the degree of deployment of SPH program elements. The survey was developed by the research team and was composed of the following three sections: (1) percent of program elements deployed, (2) percent handling equipment installed, and (3) adequacy and usage of equipment. Section 1 contained 10 elements including ceiling lifts, policy, competency evaluations, peer leader program, staff involvement in equipment selection, after actions reviews, assessments forms, routine orientation of new clinical staff, marketing program, and bariatric program. For each element, patient care areas (acute care, ambulatory care, long-term care, diagnostic, morgue, and therapy) received percent implementation scores. In section 2, percent equipment coverage was recorded for each nursing unit (acute care, ambulatory care, long-term care, diagnostic, morgue, therapy, and spinal cord injury). Finally, for section 3, the facility coordinator was asked to rate the adequacy and usage of the equipment, number of patient handling devices, number of slings, use of devices by caregivers over manual handling, and how the facility was situated to implement the program from completely disagree to completely agree (5 points).

The Program Status Report was developed by the investigators to examine human factors associated with implementing the program, and was organized into five sections: (1) facility coordinator demographics, (2) caregiver training, (3) peer leader program, (4) marketing, and (5) program support. Each section contained at least one open-ended question generally related to effectiveness. The last portion of the survey contained open-ended questions about facilitators, barriers, and customization of program implementation.

In section 1 of the Program Status Report, the survey collected data on the demographic patterns and turnover of the facility coordinator. All coordinators were asked about the implementation of their position as the facility coordinators (with 0, 25, 50, 75, or 100% implemented as anchors). The position development questionnaire included questions about the status of the job description, maintenance of position, role expectations, succession planning, and role orientation. The coordinators were also asked to rate their own effectiveness in implementing the program from extremely effective to extremely ineffective (based on a 5-point Likert scale). In section 2, they rated the caregiver training for the five patient areas (acute care, ambulatory care, long-term care, diagnostic, morgue, and therapy) with 0, 25, 50, 75, or 100% implemented as anchors. In section 3, the coordinators completed a grid based on the number of peer leaders needed, trained, vacant positions, and the number who had received annual training for each of five patient care areas. Similar to their self-rating, the coordinators also rated peer leaders for two areas: (1) position development and maintenance such as job description, role expectations, succession planning, role orientation, and whether they have selected persons to cover each shift in all care clinical, and (2) overall effectiveness of peer leaders in implementing the program. The marketing section contained two questions about the development of a marketing plan and the implementation of the plan, with 0, 25, 50, 75, or 100% implemented as anchors. In the last section, the coordinators rated the support received from 12 individual persons or groups regarding the program, for example, facility senior leadership and peer leaders, using a 5-point Likert scale from extremely supportive to extremely unsupportive.

Injury data and staff demographics were collected from VA Nursing Outcomes database, Nurse Staff Injury database, along with reports on incident records from the Automated Safety Incident Surveillance Tracking [22]. The Automated Safety Incident Surveillance and Tracking System was created in 2003 to track work-related injuries and illnesses and to serve as a data collection repository for Occupational Safety and Health Administration, VA headquarters, regional directors, and facility safety managers. We collected the 2004 (first year available) through 2011 data on MSIs associated with lifting and repositioning patients among direct care nursing occupations (nurse, practical nurse, and nursing assistant).

Nurses have the highest number of injuries in the VA and make up the greatest percentage (31% as of 2011) of workers in the VA. MSI data extraction was restricted to direct care nursing occupations and the anatomical sites of back, abdomen, and trunk. MSI incidence rate, defined as the number of injuries and/or illnesses per 10,000 full-time nursing occupation employees, is calculated as follows: Incidence rate=(N/EH)×20,000,000. In this formula, N is the number of back, abdomen, or trunk injuries; EH is the total hours worked by nursing employees (nurse, practical nurse, and nursing assistant) during the fiscal year; and 20,000,000 resulted from 10,000 equivalent full-time nursing occupation employees (working 40 hours per week, 50 weeks per year).

Bed days of care data was extracted from the National Bed Control System file, which is available from the VHA Support Service Center [22]. This dataset is designed to assist facilities by monitoring their authorized, operating, and unavailable bed capacity at a specific point in time (eg, the last day of each month or fiscal year).

The VHA’s 2011 Facility Complexity Model classification is based on seven standardized criteria [23]: (1) volume and patient case mix, (2) clinical services provided, (3) patient risk calculated from VA patient diagnosis, (4) total resident slots, (5) an index of multiple residency programs at a single facility, (6) total amount of research dollars, and (7) the number of specialized clinical services [23]. The 2011 model identified five ranking complexity levels: 1a, 1b, 1c, 2, and 3, where 1a is the most complex and 3 is the least complex.

#### Tier 2

The Peer Leader Survey was developed by the investigators and distributed to all peer leaders at each of the 17 regions included in Tier 2. The survey contained 28 items organized into following four sections: (1) demographics, (2) perceived support for the program, (3) peer leader job activities, and (4) effectiveness ratings by peer leaders (of themselves and also their facility coordinators). The nine demographic questions concerned their work, such as employment duration at this hospital, area of specialty, and length of time as a peer leader. The peer leaders rated the support received from individual persons or groups (eg, VHA senior leaders, other peer leaders) regarding the program. They were asked to indicate the number of times they had done a specific job activity during the past week, for example, demonstrating the use of patient handling equipment or dealing with a problem that arose while operating the lifting device.

The staff survey was developed by the investigators to capture information on: (1) demographics, (2) culture of safety, (3) job satisfaction, and (4) personal injury severity. The same demographic questions used in the Peer Leader Survey were repeated in the staff survey also. Two of the eight components of the Agency for Healthcare Research and Quality Hospital Survey on Patient Safety Culture [24] were used to capture the culture of safety; these included “nonpunitive response to error” and the “feedback and communication about error” dimensions. The survey has acceptable evidence for reliability and validity and is widely used in hospital settings [25].

The Stamps and Piedmont Index of Work Satisfaction Instrument was used to measure job satisfaction for nurses [26]. With permission of the instrument author, the investigators made a single modification to the survey to be inclusive of all caregiver positions and accomplished this by changing all “nursing” profession references to “in your profession” or other similar language. Past use of the Index of Work Satisfaction Instrument indicates that an SPH program resulted in increased job satisfaction for 5 of the 6 components (pay, professional status, task requirements, autonomy, and organizational policies) with statistically significant increases in both the professional status and the task requirements components [26].

To capture information on staff injury incident related to patient handling in the past 6 months, 12 questions were asked. Follow-up questions included: where they received treatment for the injury, what type of treatment they received, how many treatment visits were needed, how the treatment was paid for, how many days in total were taken off, and how many days the staff person worked on a restricted or modified duty basis. These questions were previously created for a prior study on the VA SPH program [14].

#### Tier 3

Data was collected through site visits, which included individual interviews, focus groups, observations, and taking photographs. Data collection tools for this tier included: (1) previsit clinical coordinator phone call script, (2) interview guide (individual or focus group), and (3) unit observation walk-around checklist.

The previsit clinical coordinator phone call script was used to (1) collect data on the SPH policy of the facility, (2) identify key informants and others for interviews during the site visit, and (3) identify a unit that was doing well with implementing SPH and a unit that was struggling to implement SPH. During the call, coordinators were told to recruit key informants for the site visits, from the safety committee chair, industrial hygienist, occupational medicine physician, engineering service chief, peer leaders, the nurse executive, and nurse mangers. Altogether, they were asked to recruit 27-34 individuals for each site visit. A list of potential candidates was emailed to the facility coordinator. The interview guide for individuals and focus groups contained a general overview of procedures, ground rules, and confidentiality. Interview questions asked about roles in the program, what was working well and not, barriers and facilitators to program implementation, strategies to overcome barriers or customize the program, and surprises in implementation, sustainability, and support. Peer leaders, facility coordinators, managers, and administrators were further prompted to explain their roles and reasons for choosing those roles. Prompts and storytelling techniques were used to stimulate the discussion. For example, staff members were asked to describe a recent interaction with another staff member regarding the program.

The unit walk-around checklist included general questions for staff about their perceptions of the SPH program and targeted questions on the number of beds, number of ceiling lifts, method of equipment handling, training received, sling storage, equipment maintenance, and comfort in using equipment. Open-ended questions addressed the availability of equipment, supervisory support, protocols on the unit, and who would they go to for help regarding SPH and when.

### Data Collection Procedures

Data collection for Tier 1 included extractions from several VA extant databases including Veterans Affairs Nursing Outcomes database, Automated Safety Incident Surveillance and Tracking System, Bed Cube, and the Human Resources Employee Cube. Data were extracted using ProClarity Microsoft software [27] and exported into a spreadsheet and Statistical Analyses System [28] software for analysis. All three facility surveys (ie, Milestone Questionnaire, Program Dose Survey, and Program Status Report) were sent to each facility coordinator who then returned completed surveys using a traceable mail service, an exclusive fax, or an encrypted email within the VHA firewall.

The survey instructions to the facility coordinators explained that the hospital or regional director might want to see responses to the Milestone Questionnaire, but responses to the Program Status and Program Dose questionnaires should not be shared.

In Tier 2, the local site investigators sent emails to invite the peer leaders of their facilities asking them to participate in the survey. A separate email invitation was sent to the frontline staff to participate in the staff survey. All recruitment materials and survey instructions stated that the surveys were completely voluntary, that no rewards would be given for participation, and that the surveys would remain anonymous. Participants in both Peer Leader and Staff surveys were offered the options to fill out the survey either online or using paper-and-pencil version of the survey. The same security procedures were followed for either option as described in Tier 1.

For Tier 3, we collected data during previsit phone calls with facility coordinators, 1- to 2-day visits to facilities and during post-site visit activities. Using a scripted guide, during previsit phone calls, investigators planned site visits with the facility coordinator, requested SPH policies, and finalized logistics of the site visit. During the site visits, experienced investigators conducted 1-hour-long semistructured interviews with key informants (individuals and focus groups) using the interview guide. During the unit walk-arounds, the facility coordinator or other staff escorted investigators to selected units where they made observations, conducted informal interviews with caregivers, and photographed places and persons to capture activities illustrative of key concepts and local innovations. After the site visit, investigators conducted debriefings to note significant observations, identify preliminary conclusions and gaps in data, and determine if changes in approaches to data collection or in the use of data collection instruments were warranted. Post-site visit activity also included document review of the facility SPH policy.

### Data Quality: Missing Data

Missing data can be a potential problem in any large-scale longitudinal study, particularly those that rely, in part, on administrative data systems. To minimize missing data in Tier 1, the project manager maintained a database to track incoming data and followed up by phone calls and emails to increase response rates. The milestone survey was an administrative requirement associated with program funding; therefore, high response rate was expected.

All survey data were scanned into a database using Teleform [29]. Efforts were made to identify and correct errors in data collection, coding, and entry. Keystroke error rate computed on a 10% sample of the data was found to be less than 2%, which ensures accuracy. Data cleaning was initiated early during the data collection; this process allowed us to limit propagation of any systematic error during subsequent stages of data handling.

To ensure data quality of interviews, experienced focus group facilitators and individual interviewers conducted all interviews. Data collection and analysis ran concurrently in the interviews, that is, feedback was obtained as conclusions were drawn; an assistant recorded notes of discussions during the focus groups; audio tape recordings of interviews were used to ensure no material was missed during analysis; and analysis was conducted by two investigators. The data record of Tier 3 included notes from the pre-site visit interviews and unit walk-around observations, field notes written by site visitors, transcripts of the interviews and focus groups, and photographs taken during the unit observations for all site visits.

### Statistical Analysis Plan

#### Overview

The study used a prospective cohort of facilities, in observation design with up to five waves of data collection points over time. Because the SPH program was already implemented by all VAMC facilities before the evaluation study was established, there will be no intervention-control groups for testing comparative effects of the SPH program. Therefore, we propose a standard approach to investigating causal effects in designs without a control group. That is, our design can yield strong causal inferences mainly by reducing the plausibility of alternative explanations for the program effects [30]. Given the multicomponent nature of the SPH program coupled with a 3-tiered data collection process, we specified our hypotheses in general terms initially. Simplified and more specific version of each general hypothesis for each tier is described in the following text.

#### Tier 1

Summary scores for each program component will be produced as a continuous measure for each facility at each data wave, and dose-response approach to analysis will be used. Where appropriate, the scores from Tier 1 survey data will be calculated as average over time to obtain a single score per facility before they are used as predictors in a model. To investigate our primary outcome (ie, injury incidence rate of caregiver patient handing), a more specific H2 is: after accounting for contextual factors (Bed Days of Care, Facility Complexity, and baseline MSI incidence rates), higher scores on SPH implementation, strength, and SPH uptake measures will be significantly associated with (1) lower 2011 MSI incidence rates and (2) greater decline of MSI incidence rates over the study period. The more specific H3 can be explained as follows: the effects of contextual factors on MSI incidence rates will be moderated by SPH implementation and SPH uptake. A multiple regression model of 2011 MSI incidence rates with contextual factors as predictors will test H1. For H2a, H2b, and H3, SPH components and contextual factors will be included as predictors, respectively, in (1) a multiple regression model of 2011 MSI incidence rates, (2) a growth curve model of repeated observations of MSI incidence rates over time, and (3) additional testing of two-way interactions between SPH components and contextual factors in the regression models. The models will evaluate the direct effects of the SPH program components on MSI rates, as well as moderation of the effects of contextual factors by program components. The growth model will facilitate a greater understanding of the individual facility differences in change in MSI incidence rates over time. Parallel hypotheses will be similarly tested for handling-related patients’ injuries and costs outcomes.

To accommodate both linear and nonlinear relationships in the data, the Generalized Additive Model method available in the R package *mgcv* [31] will be used. A major strength of Generalized Additive Model is that it employs scatter plot smoothers which are nonparametric techniques that define data relationships in a flexible way, thereby relieving the user from the need to search for the appropriate transformation for each predictor [32-34]. The coefficients of each SPH program component and its interaction(s) will capture the magnitude and direction of that SPH program component effects, adjusted for other covariates in the model. For the overall SPH program effects, we will identify optimal subset of the components that significantly explains the greatest amount of unique variance (estimated as *R* ^2^) in the outcome in the most parsimonious way, that is, variation explained in addition to the proportion already explained by a base model containing only non-SPH program variables.

A two-level latent growth curve model will be specified (ie, time at level 1 nested within facility at level 2). The time trends of MSI incidence rates (level 1) will be characterized by estimated growth parameters for individual facilities. Simultaneously, the growth parameters will be modeled as dependent variables that are predicted by facility-level variables (level 2), for example, by contextual factors and SPH component scores. The estimated growth parameters including intercept, linear slope, and quadratic slope will, respectively, approximate initial MSI incidence rate, rate of decline of MSI incidence rate, and acceleration of decline of MSI incidence rate. Only the first two parameters need to be estimated and modeled if prior graphical examination reveals linear trends, or if the estimated quadratic parameter is not statistically significant. Within the structural equation model framework, the contextual factors will be added first to the model as plausible alternative explanatory variables for the growth factors, followed by SPH program components (eg, SPH uptake). This will allow SPH components to explain systematic patterns of MSI incidence rates over time across facilities while accounting for alternative sources of variation in these patterns. We will explore two options to incorporate SPH program scores in the growth model. The first option will use each program component score averaged over time per facility as a static (time-invariant) predictor variable. The second option will apply the repeated assessment scores of each component as a dynamic (time-varying) predictor variable having different values at different time points. To investigate the meditational process of SPH program effects, we will test a number of specific H4: for example, higher levels of Peer Leader effectiveness (predictor) will lead to increased use of equipment (mediator), which will cause reduction in MSI incidence rate (outcome); and the proportion of the total effects of predictor on outcome that is mediated can also be determined using a single mediator model [35]. The growth and mediation modeling will be performed using Mplus Version 6 statistical application [36].

#### Tier 2

Since individual facilities but not persons can be linked across the four assessment occasions at Tier 2, two approaches can be adopted to analyze both Staff and Peer Leader surveys: (1) choosing a single time point (eg, Feb 2011 job-satisfaction data to which all 17 facilities contributed; N=1643 staff), and (2) analyzing change over time using data aggregated at the facility level (N=17 facilities). Consistent with our global H2, here we hypothesize that after accounting for contextual factors, higher scores on SPH implementation strength and/or SPH uptake measures will be significantly associated with (1) higher staff job satisfaction and (2) steeper positive trend in staff job satisfaction over the study period. In keeping with H3, we also hypothesize that contextual factors (eg, culture of safety) will be significantly associated with SPH program implementation strength (eg, amount of training).

In the first approach, a multilevel analysis will be appropriate given the nested structure of the data (individuals are nested within facility). Therefore, to test a variable-specific H2(a), a two-level regression model will be used to examine Feb 2011 staff job satisfaction (individual level 1) as a function of predictors both at the individual level (eg, staff demographics, professional experience) and at the facility level (eg, SPH program implementation scores, Bed Days of Care). For another specific H2(b), a growth model will test the predictive effects of the scores of SPH program implementation (averaged over time) on the trajectory of staff job satisfaction as described by growth parameters including intercept (initial status) and linear slope (rate of change), while adjusting for other covariates. To test hypothesis H3, a regression model of program implementation strength (eg, policy development) will be fitted to include contextual factor (culture of safety) aggregated at facility level as a predictor. All modeling will be performed in Mplus [36].

#### Tier 3

In the qualitative tier, all electronic interview and focus group files, field notes, documents, and photographs were transcribed verbatim or scanned and stored on a secure VA server with access only by the research team. All files were loaded into the qualitative computer analysis software program ATLAS.ti to systematically develop a code book that catalogued and organized defined codes. Qualitative data were analyzed using a content analysis approach that used memos, process mapping, and diagramming to describe, categorize, and connect the data to determine common themes. For this purpose, analysts (1) assigned first level codes to units of meaning, (2) synthesized codes into complex categories, (3) compared and contrasted the categories to identify relationships across categories, (4) grouped categories into a taxonomic structure that described the data set, and (5) linked representative sections of text to the categories to identify salient quotes that illustrated the codes and constructs and that supported the coding decisions. Multiple sites, interviews, focus groups, and observations allowed for methodological triangulation, thereby increasing likelihood of credible findings.

Logistically, Drs. Besterman-Dahan and Elnitsky led the qualitative data analysis and Dr. Powell-Cope provided consultation as needed. In later stages of the project, additional analysts joined the qualitative team. Initially, the team members compared and contrasted perceptions of key findings following interviews, focus groups and observations at individual sites. The analysis strategy of the project was as follows: (1) data analysts reviewed the first few transcripts and developed codes independently, (2) they reviewed their work together and through consensus agreed on codes and definitions, (3) they continued to double code transcripts making memos and including field notes where relevant, and (4) after 90% agreement was attained, they coded transcripts independently using the common codebook. Every fifth coded transcript they met to review randomly selected portions for agreement, and discrepancies were discussed and decisions were made jointly to determine whether new codes were needed. Data analysts met regularly to review ongoing coding results and resolve coding issues.

### Ethics Approval

Approval for the study was obtained by the VA Research and Development Committees and the associated Institutional Review Boards at the evaluation center facility and all facilities participating in Tiers 2 and 3. Approval for Tier 1 data collection, with a waiver of informed consent was sought and granted from the Department of Research and Development, James A Haley Veterans Hospital and from the Institutional Review Board, University of South Florida, in Tampa Florida. For Tiers 2 and 3 data collection, permission from each site respective VA Research and Development Department and Institutional Review Board were sought and obtained. To maintain anonymity, we received waivers of written informed consent for Tier 2 Peer Leader and Staff surveys. We obtained written informed consent for all interviews conducted and all photography of persons in Tier 3.

## Results

All three tiers of data for this study have been collected. We are now in the analyses and writing phase of the project, with the possibility for extraction of additional administrative data. This study evaluates the effects associated with the national implementation of a comprehensive SPH program that was implemented nationally in all 153 VAMCs to reduce patient handling injuries.

## Discussion

### Overview

To our knowledge, this is the largest evaluation of an SPH program in the United States. The direct cost of treating an average back injury case is US $19,000, with serious cases involving surgery costing as much as US $85,000 [37]. The large numbers of nursing professionals at risk of back injury in the VA system is the rationale for this research. With the data collected, as the administrative data, we have the opportunity to ascertain the impact of the program on nursing back injuries and other outcomes such as job satisfaction. This could result in a reduction of direct cost due to the potential reduction in the number of injuries. Another benefit of the research is the ability to examine program implementation and sustainability. The VA is continuously at the forefront of program implementation for patients and the health care workers. This study will look at links between the outcomes and the role of moderating variables, such as program dose and organizational factors on the outcome. The qualitative sections will answer contextual questions about barriers and sustainability of the program.

### Limitations

However, there are some potential limitations in this study. The lack of experimental groups for comparison is a limitation of the present design, particularly at Tiers 1 and 2. Another major concern is that the potential SPH program effects may be compounded with cohort effects (changes and variations occurring irrespective of intervention as the cohort of facilities moves through time). However, these limitations have been partially addressed by the adoption of dose-response approach and do not constitute a serious threat to the study design and inferences based on the results.

A major strength of this observational study design is that all existing 153 VAMCs implemented the program and are included in Tier 1. Therefore, population sampling bias in relation to tests of program effects is not a concern. Moreover, unlike the typical experimental and controlled designs, our nonexperimental one-cohort design in which repeated data points capture differential program implementation rates across all VAMC facilities and over time better approximates a “real-world” situation. Although designs without control groups make it difficult to know what would have happened without an intervention, the differential program implementation or intervention exposure rates (across facilities and over time) allow us to tap into some information about what might have happened to facilities had the SPH program not been implemented in terms of degree and timeliness. Also, the longitudinal setting for this study establishes a temporality critical for establishing a cause-and-effect relationship among key variables, while also accounting for competing time effects (eg, as intrafacility change at level 1 of the growth model). To enhance external validity of findings from Tier 2 data, some degree of control was instituted for the sample selection. In this part of the study, 17/21 (81%) regions were represented.

### Conclusions

In conclusion, VA-wide implementation of the SPH program afforded the opportunity for rigorous evaluation in a naturalistic context. In the process, we are posed to contribute to implementation science in health care by linking program dose and quality to outcomes. Outcomes will be useful for VA operations for policy and resourcing decision making, and to health care outside of the VA in decision making about SPH programming.

## Abbreviations

MSI: musculoskeletal injuries
SPH: safe patient handling
VAMC: Veterans Administration Medical Center
VHA: Veterans Health Affairs
